# Editorial for the Special Issue on Micro/Nanofluidic Devices for Single Cell Analysis, Volume II

**DOI:** 10.3390/mi12080875

**Published:** 2021-07-26

**Authors:** Tuhin Subhra Santra, Fan-Gang Tseng

**Affiliations:** 1 Department of Engineering Design, Indian Institute of Technology, Chennai 600036, India; 2 Department of Engineering and System Science, National Tsing Hua University, Hsinchu 300044, Taiwan; fangang@ess.nthu.edu.tw

The functional, genetic, or compositional heterogeneity of healthy and diseased tissues promotes significant challenges to drug discovery and development [1,2]. Genetically identical cells may exhibit phenotype heterogeneity, which is of particular importance for tumor metastasis, stem cell differentiation, and drug resistance [3]. Such heterogeneities impede accurate disease modeling and can mislead the elucidation of biomarker levels, and may misguide patient responses to particular therapies [1,2,3]. Nevertheless, cellular heterogeneity has remained unexplored for a long time as former studies mainly focused on cell manipulation and analysis at the bulk scale, providing the average interpretation of the results. The complex nature of cells has been the long-standing motivation for developing the tools for single-cell transcriptomic, genomic, and multiplex proteomic analyses [1,4,5,6]. However, the traditional biological tools, including petri-dishes and well-plates, technically limit micron-scale single-cell manipulation and analysis. Additionally, the use of low concentrations target biomolecules introduces additional challenges in this field. Therefore, single-cell research provokes the access of modern technologies to address single-cell functionalities with high-throughput efficiency [1,4,7].

Single-cell technologies are beneficial for the studies of scarce cells [1,4,8,9]. For example, circulating tumor cells (CTCs) are rare, such as one in the background of 10^7^ normal blood cells. Detecting and characterizing these cells could help and explore the underlying cause of cancer spreads and be very useful in developing efficient and targeted therapies [10,11]. Usually, single cells [1,4] have been isolated by multi-well plates in most biological labs, which provides low efficiency, and significant labor strength is needed. Another option is robotic liquid handling workstations that reduce labor intensity but are pretty expensive to install in the lab. The standard techniques, such as flow cytometry and laser scanning cytometry, can rapidly screen fluorescent-labeled cells in a flow, and these have been used for single-cell analysis for a long time [12,13]. Flow cytometry is an automatic technique for multiple detections and sorting of single cells. However, the instrument is expensive, bulky and mechanically complicated. It requires large sample volumes and analyzes cells at a one-time point. Hence, flow cytometry cannot provide continuous variation in the cell dynamics.

On the other hand, micro/nanofluidic devices have emerged as a potential platform with advanced technologies for single-cell manipulation and analysis in the last two decades. Micro/nanofluidic devices have many unrivaled advantages over conventional techniques [14,15,16,17]. They can manipulate and control fluids in the range of micro to pico-liters, thus reducing sample loss, providing susceptible analysis in the miniaturized microfluidic systems [14,15,17]. The micro/nanofluidic devices are not only designed and fabricated to fulfill the needs of various single-cell manipulation, separation, trapping isolation and lysis, but also used for electrical, mechanical, optical, biochemical characterization, as well as for therapeutic and diagnostic purposes [18,19,20,21,22,23,24,25,26]. Figure 1 shows worldwide microfluidic single-cell-related article publications in the last two decades, and it indicates the high demand of microfluidic devices for single-cell analysis and applications, evidenced by the increase in the number of scientific publications in each year. The micro/nanofluidic devices expedite remarkable high-throughput parallel manipulation and analysis of single cells, providing more accurate statistical results than bulk analysis and having a meaningful interpretation. Moreover, multifunctional devices can be integrated on the same chip to make it automatic, eliminating possibilities of contamination and error-free operations. Additionally, fluorometry, mass spectroscopy, and fluorescence microscopy can be integrated with microfluidic systems for achieving deeper insights into single-cell morphology and functionalities. Such steps can pave new avenues in this exciting field. This Special Issue of *Micromachines* entitled “Micro/nanofluidic devices for single-cell analysis” encompasses the recent advancements in single-cell analysis using micro/nanofluidic devices.

Hochstetter [27] briefly reviewed single-cell separation, diagnostics, and analysis using recent advancements in lab-on-a-chip technologies. Moreover, they reviewed the potentials, limitations, future prospects, and applications of microfluidic technologies, especially concerning the funding outlook and field requisition of the chips.

The measurement of sample flow velocity is essential for controlling the cell sorting time and reconstruction of image analysis. Sawa et al. [28] reported on-chip microparticle size and velocity assessment by using differential image analysis of single-shot two-wavelength. While the microparticles run via an image flow cytometer, they are irradiated by two different lights with different irradiation times simultaneously. For each wavelength of light, the images of the same microparticle were captured in a single shot. The velocity is calculated by comparing these two images: the difference of the particles’ elongation divided by the difference of irradiation time. These accurate velocity and shape measurements can improve the cell sorter technique and the imaging flow cytometry to diagnose cells.

The high-throughput in vivo cellular microenvironment can permit us to investigate cellular function in detail. Nagai et al. [29] developed a parallel single-cell manipulation using a micronozzle array compacted with a bidirectional electrokinetic micropump. The polydimethylsiloxane (PDMS) micro nozzle array combined with bidirectional electrokinetic pumps are operated by using DC-biased AC voltages. Single HeLa cells were transported to the nozzle holes. After applying voltage, adequate electroosmotic flow occurs outside the nozzle array and manipulates the single-cell simultaneously.

Li et al. [30] demonstrated the hydrodynamic cell stretching and microsieve filtration, which can express the link between genetic, phenotypic, and treatment to the cellular deformability. This cellular deformability has shown the correlation between metastatic cancer cells and invasiveness. In-depth studies on single-cell hydrodynamic stretching can correlate mechanical characteristics of cancer cells with genetics and phenotypes. It helps to distinguish the differential deformability of cell models toward promoting drug treatment, EMT, and invasiveness, thus strengthening our knowledge on the fundamentals of cancer progression.

The electrical properties of the cells cannot be measured effectively by conventional techniques. Liang et al. [31] proposed a microfluidic-based constriction channel to characterize the electrical properties of a single nucleus. The device can isolate and trap single nuclei at the microfluidic channel without any pipette tips for electrical measurements. Their technique can classify cell type and cell status evaluation through bioelectrical markers of cell nuclei. Here, the authors studied the effect of membrane capacitance on the estimation of nuclear electrical properties and compared it with electrorotation.

Sengul and Elitas [32] presented sensitive, label-free, and specific, single-cell electrochemical properties using a microfluidic device. They fabricated a 3D carbon electrode array-based device and showed deformation measurement of U937 monocytes and dielectric movement and U937-differentiated macrophages in a less conductive medium. Using their technique, the cell damage caused by aggressive shear forces can be measured, and cells can be used for further downstream analysis. Moreover, these results also revealed that dielectric mobility and deformation could be exploited as an electromechanical biomarker to recognize differentiated cell populations from their progenitors.

The same group [33] investigated the impact of macrophages on glioma cell behavior by using a microfabricated cell culture platform. They quantified motility, migration, morphology, proliferation, and deformation characteristics of glioma U87 cells at the single-cell resolution to unveil biomechanical heterogeneity. They could quantify the mechanophenotypic properties of glioma cells by using their microfluidic device.

Cytokine secretion has a tremendous impact on clinical diagnostics. Zhu et al. [34] reported Cytokine secretion detection at the single-cell level and real-time monitoring using localized surface plasmon resonance (LSPR). The authors developed a microwell chip with cyclo-olefin-polymer (COP) film imprinted with gold-capped nanopillars for Interleukin 6 (IL-6) detection at the single-cell level. The trapped cell secret cytokine was analyzed by using the spectrum analyzer. This fabricated device facilitates real-time monitoring that can monitor the biological variation of the tested single-cell viability.

The CTCs can be considered a substitute approach for tissue biopsy, and it able to provide tumor-derived and germline-specific genetic variations. The analysis of the CTCs at a single-cell level can enable in-detail tumor heterogeneity exploration and individual clinical assessment. Xu et al. [35] demonstrated CTCs isolation and clinical application by using a microfluidic chip integrated with a micropore-arrayed filtration membrane. The device has the ability to provide CTCs isolation with high efficiency, throughput, and minimal damage of the cell. Moreover, the device can detect a positive detectable rate of 87.5% CTCs from lung cancer patients. This detection method can be a promising tool for cancer research and the accomplishment of CTCs analysis for routine clinical practice.

In conclusion, this Special Issue entitled “Micro/nanofluidic devices for single-cell analysis” not only covers single-cell manipulation, separation, diagnostics but also it discussed single-cell mechanical, electrical, and electrochemical characterizations and their analysis. Moreover, this Special Issue elaborates on cellular heterogeneity characteristics, Cytokine secretion detection, circulating tumor cell (CTCs) isolation, and clinical applications.

## Figures and Tables

**Figure 1:**
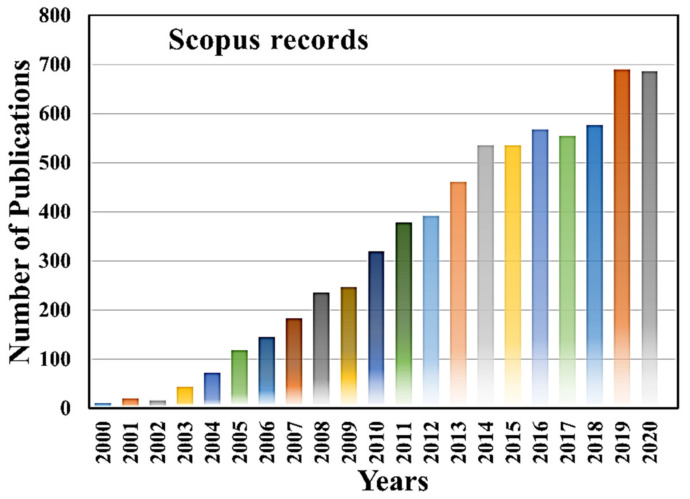
Year-wise microfluidic single cell scientific article publication. The data are adapted from Scopus records until 2020.
